# Liposomes Loaded with Hydrophobic Iron Oxide Nanoparticles: Suitable T_2_ Contrast Agents for MRI

**DOI:** 10.3390/ijms17081209

**Published:** 2016-07-27

**Authors:** Raquel Martínez-González, Joan Estelrich, Maria Antònia Busquets

**Affiliations:** Department of Pharmacy, Pharmaceutical Technology and Physical Chemistry, IN^2^UB, Faculty of Pharmacy, Avda Joan XXIII, 27-31, 08028 Barcelona, Spain; raquelmartigonza@gmail.com (R.M.-G.); joanestelrich@ub.edu (J.E.)

**Keywords:** magnetoliposomes, hydrophobic Super Paramagnetic Iron Oxide Nanoparticles (SPIONs), Magnetic Resonance Imaging (MRI), relaxivities, contrast agents

## Abstract

There has been a recent surge of interest in the use of superparamagnetic iron oxide nanoparticles (SPIONs) as contrast agents (CAs) for magnetic resonance imaging (MRI), due to their tunable properties and their low toxicity compared with other CAs such as gadolinium. SPIONs exert a strong influence on spin-spin *T*_2_ relaxation times by decreasing the MR signal in the regions to which they are delivered, consequently yielding darker images or negative contrast. Given the potential of these nanoparticles to enhance detection of alterations in soft tissues, we studied the MRI response of hydrophobic or hydrophilic SPIONs loaded into liposomes (magnetoliposomes) of different lipid composition obtained by sonication. These hybrid nanostructures were characterized by measuring several parameters such as size and polydispersity, and number of SPIONs encapsulated or embedded into the lipid systems. We then studied the influence of acyl chain length as well as its unsaturation, charge, and presence of cholesterol in the lipid bilayer at high field strength (7 T) to mimic the conditions used in preclinical assays. Our results showed a high variability depending on the nature of the magnetic particles. Focusing on the hydrophobic SPIONs, the cholesterol-containing samples showed a slight reduction in *r*_2_, while unsaturation of the lipid acyl chain and inclusion of a negatively charged lipid into the bilayer appeared to yield a marked increase in negative contrast, thus rendering these magnetoliposomes suitable candidates as CAs, especially as a liver CA.

## 1. Introduction

Magnetic resonance imaging (MRI) is one of the most powerful noninvasive imaging techniques in diagnostic radiology, due to its high soft tissue contrast, spatial resolution, and penetration depth [1,2,3,4]. The magnetic fields employed MRI for clinical diagnosis are of 3 T, but preclinical studies with small animal models, which need the highest possible resolution, rely on very high field strengths (>7 T). Despite the relatively high quality of the MR images of the soft tissues, in some cases it is not possible to have enough image contrast to diagnose the pathology of interest. In these cases, a contrast agent (CA) is needed. The CA improves the contrast-to-noise ratio in MRI by shortening the spin-lattice *T*_1_ and/or spin-spin *T*_2_ relaxation times of the water protons within the tissues/regions of interest, thus enhancing the image contrast. Therefore, what is imaged is not the agent of contrast, but rather its effect on the relaxivity of the adjacent water protons, predominantly through the dipolar interaction [5]. The increase of MRI contrast produced by the magnetic nanoparticles is dependent on their composition, size, surface properties, and of the extent of aggregation in the biological milieu [6,7]. The efficiency of a CA depends on its *r*_1_ and *r*_2_ relaxivity as well as their ratio. The higher the ratio of *r*_2_/*r*_1_, the better the efficiency of a *T*_2_ CA and vice versa for a *T*_1_ CA [8,9]. Paramagnetic substances, such as gadolinium (Gd), are positive contrast agents (*T*_1_ CA). They enhance the MR signal intensity. However, they present two clinical limitations: Gd complexes have a certain degree of toxicity, and their efficiency at higher magnetic fields decreases. Due to these limitations, the research focus has shifted to negative CA such as superparamagnetic iron oxide nanoparticles (SPIONs). As compared to gadolinium compounds, SPIONs show the advantages of tunable size and shape, as well as possibility of surface modification and more effectiveness at lower concentrations because of their superparamagnetic property. SPIONs decrease the MR signal intensity of the regions where they are delivered and thus those regions appear darker in the image. Magnetite (Fe_3_O_4_) and maghemite (*γ*-Fe_2_O_3_) are two types of SPIONs, easily prepared in the laboratory, that have been used for biomedical applications since they meet the following criteria: (1) chemical stability under physiological conditions; (2) low toxicity and (3) high magnetic moments [10]. There are several formulations of SPIONs that have been approved by the U. S. Food and Drug Administration (FDA) and the European Medicines Agency (EMEA) for clinical use as MRI CA [11]. However, the majority of the approved compounds are, at present, out of the market [12]. The SPIONs for biomedical applications are usually prepared by hydrolytic methods, mainly by alkaline co-precipitation of stoichiometric amounts of Fe(II) and Fe(III). Such methods yield hydrophilic but usually polydisperse nanoparticles, and, in consequence, the nanoparticles can form large agglomerates in physiological media. Therefore, small well-defined SPIONs with a narrow distribution are of great interest, since magnetic properties change drastically with particle size. The thermal decomposition of metal precursors in organic media, a non-hydrolytic method, produces high-quality SPIONs with uniform size and high crystallinity. However, these SPIONs are hydrophobic. To achieve the necessary stability in aqueous media, the modification of their surface is required. Several methods based on the modification of the surface have been developed; ligand exchange with water-dispersible ligands and the encapsulation of ligands are two of the most representative strategies of such modification [7,13].

Considering the fact that phospholipids present several advantages such as biocompatibility, biodegradability, and reduced toxicity, liposomes can be used as a coating for the SPIONs. Combining nanoparticles with liposomes is a highly elaborative methodology in the emerging fields of nanomedicine and nanobiotechnology, since liposomes can carry on either hydrophobic or hydrophilic nanoparticles. SPIONs can be hybridized with liposomes to get magnetoliposomes (MLs; for a review on MLs, see [14,15]). MLs have been used as CA for molecular imaging and as a theranostic tool [16,17,18,19,20], but mainly as an efficient MRI CA with enhancing *T*_2_ contrast, although some groups have combined the *T*_1_ and *T*_2_ MRI CA in an unique system to obtain bimodal CA [16].

The magnetic properties of SPIONs depend on various factors, such as size, shape, composition, and crystallinity [11]. In this way, the proton relaxivity shows a strong dependence on the particles size. Moreover, according to the Koening–Kellar model [21] the types of surface functionalization and hydrophilicity influence the longitudinal (*r*_1_) and the transversal (*r*_2_) relaxivities. Therefore, the coating of magnetic nanoparticles greatly modifies the MRI contrast, either hampering the diffusion of water molecules, or forming hydrogen bonds with water molecules, thus increasing the residence time of water [22]. Hence, the measured proton relaxation rates depend strongly on the hydrophilic nature of the coating layer.

Since liposomes can be made with different lipid formulations and can present several sizes and physical structures depending on the method of preparation, the coatings and structures interacting with SPIONs will be different.

To improve the knowledge about the magnetic relaxation associated with the contrast produced by MLs, we have studied the impact that the sonication process has on the relaxivity of liposomes obtained by this method. To achieve this, we have used hydrophilic and hydrophobic magnetic particles encapsulated in liposomes. Liposomes were made of six formulations, differing in the fatty-acid chain length, the presence or absence of cholesterol (CHOL), and the presence or absence of negative charge (afforded by phosphatidylserine, (PS)). After sonication, the relaxivity properties of such hybrid nanoparticles were determined at 7 T.

## 2. Results and Discussion

### 2.1. Characteristics

Size of different samples of MLs was determined by dynamic light scattering (DLS). A single population (monomodal distribution) constituted all MLs samples, with a *z*-diameter ranging from 220 nm to 335 nm. Polydispersity index was between 0.200 and 0.300. The size and polydispersity of such populations are strongly dependent on the sonication process. For making the comparison among the different samples possible, the conditions of the sonication process were kept invariably constant. After gel exclusion chromatography (GEC) purification, the lipid and the iron content were determined and the encapsulation efficiency was calculated as *μ*mol of iron by mmol of lipid (Table 1). The volume accessible to the ferrofluid can explain the great difference observed in the encapsulation efficiency of liposomes containing hydrophilic or hydrophobic ferrofluid. While hydrophilic ferrofluid can be distributed either inside the aqueous interior of liposomes or in the external medium, the hydrophobic one can only be located in or near the lipid double layer. Consequently, the hydrophilic ferrofluid is encapsulated in liposomes to a lesser extent than the hydrophobic magnetic core. This also implies that the GEC purification is applied exclusively to the liposomal formulations containing hydrophilic nanoparticles. 

On the other hand, the number of magnetic nanoparticles encapsulated in each liposome *N* can be calculated with the data of the final concentrations of magnetite and lipid (Table 1). *N* was determined from the ratio of the total number of magnetic nanoparticles *N*_MNP_ and lipid vesicles *N*_VES_.

(1) $$N=\frac{N_{\text{MNP}}}{N_{\text{VES}}}$$

*N*_MNP_ and *N*_VES_ have been obtained from nanoparticle size and concentration, respectively through the following equations:

(2) $$N_{\text{MNP}}=\frac{M_{\text{NP}}}{d_{\text{NP}}V_{\text{NP}}}$$

(3) $$N_{\text{VES}}=\frac{N_{\text{VOL}}}{N_{\text{lipid}}}$$

where *M*_NP_ is the total mass of Fe_3_O_4_ as obtained from colorimetric determination, *d*_NP_ is the density of magnetite (*d*_NP_ = 5.1 g·cm^−3^). *V*_NP_ is the volume of a single nanoparticle taking a diameter of 5 nm and considering nanoparticles as perfect spheres (*V*_NP_ = 65.45 nm^3^).

Assuming that liposomes are unilamellar, for a large spherical liposome higher than 200 nm, the inner and the outer layers have the same surface and thus contain the same amount of lipids. From the average radius of each liposomal sample, the total area can be calculated. Dividing this total area by the cross-sectional area of 1,2-Dioleyl-sn-glicerol-3 phosphatidylcholine (DOPC) molecules (*a*_o_ = 0.72 nm^2^) [23], 1,2-Dimyristoyl-sn-glicerol-3 phosphatidylcholine (DMPC) molecules (*a*_o_ = 0.59 nm^2^) [24], PS molecules (*a*_o_ = 0.70 nm^2^) [25] and CHOL molecules (*a*_o_ = 0.35 nm^2^) [26], the number of lipid molecules per vesicle (*N*_lipid_) can be calculated. An average cross-sectional area was used when liposomes were formed by more than one lipid (Table 1). Multiplying the lipid concentration of samples after GEC step by the Avogadro constant, the number of total lipids molecules (*N*_VOL_) by unit of volume can be obtained. From *N*_MNP_ and *N*_VES_, the number of encapsulated magnetic particles by liposome can be determined (Equation (1)).

**Figure 1:**
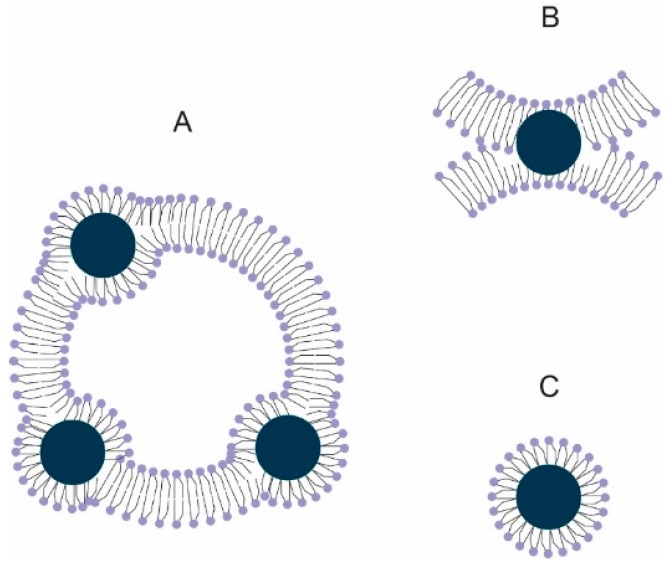
Location of the hydrophobic magnetic particle in the liposome: (**A**) Projected out of the bilayer; (**B**) embedded within neighboring bilayers and; (**C**) formation of micelle-like assemblies. Hydrophobic nanoparticles are represented by a dark blue circle whereas the phospholipids are the light blue parts (polar head joined to a two acyl chains). In a water milieu, phospholipids can form bilayers (**A**,**B**) or monolayers (micelle-like-assemblies as depicted in **C**).

Table 1 shows that the number of encapsulated magnetic particles is higher in liposomes containing hydrophobic particles than those encapsulating hydrophilic ones. When hydrophilic nanoparticles are used, their size is increased by the presence of the attached water molecules leading to an average hydrodynamic diameter of 20 nm. In consequence, the number of encapsulated nanoparticles inside the aqueous volume of a liposome is limited. Moreover, this encapsulation is based on random trapping by bilayer membrane closure. Thus, sorting as a function of intravesicle loading is usually a tricky problem. In the case of hydrophobic nanoparticles, these have a higher affinity to the acyl-chains confined in the hydrophobic interior of the phospholipid bilayer. However, we must take into account that the magnetic particles are 5 nm in size, and the average thickness of the bilayer ranges from 3.5 to 5 nm. For this reason, hydrophobic nanoparticles can sometimes project out of the bilayer (Figure 1A) or be embedded within two neighboring bilayers (Figure 1B). A third possibility is the formation of micelle-like assemblies (Figure 1C) by adsorption of phospholipids on the nanoparticle. The formation of the original MLs followed this strategy [27], although the size of the nanoparticles used was higher. A size of 5 nm, corresponding to the employed nanoparticles, involves a high curvature, and this would induce the creation of a large free volume between the acyl-chains of the monolayer. As can be observed in microscopy images, no free particles are observed. Hence, we only contemplated the two first possibilities. The embedding of a hydrophobic particle in a hydrophobic membrane is energetically favorable since it is the difference between a favorable Gibbs energy change produced by moving a hydrophobic particle from pure water into the bilayer and the energy needed to deform the hydrophobic membrane [28,29].

**Figure 2:**
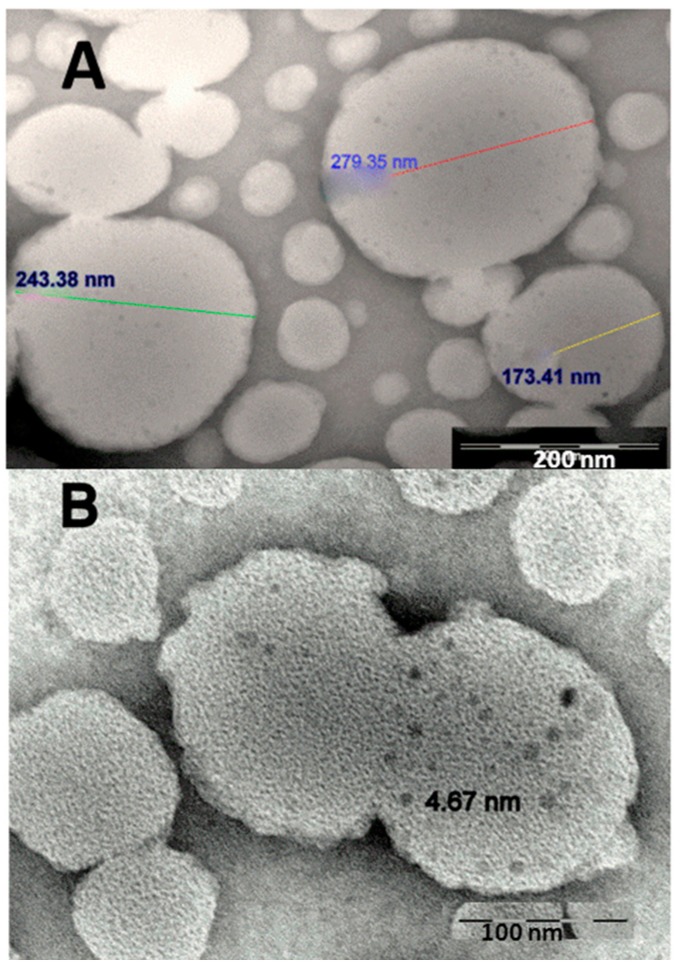
TEM images of DMPC liposomes encapsulating hydrophobic magnetic nanoparticles (MNPs). (**A**) The size of liposomes is indicated (scale bar: 200 nm); (**B**) Magnetic nanoparticles of an average diameter of 5 nm are visualized in the DMPC liposomes (scale bar: 100 nm).

In Figure 2 TEM images of ML structures are showed. Some liposomes are in close contact, perhaps by merging of neighboring bilayers. Bilayer merging was probably driven by the interfacial activity of the nanoparticles, the energy gain by a hydrophobic surface partitioning from water into a hydrophobic environment.

**Figure 3:**
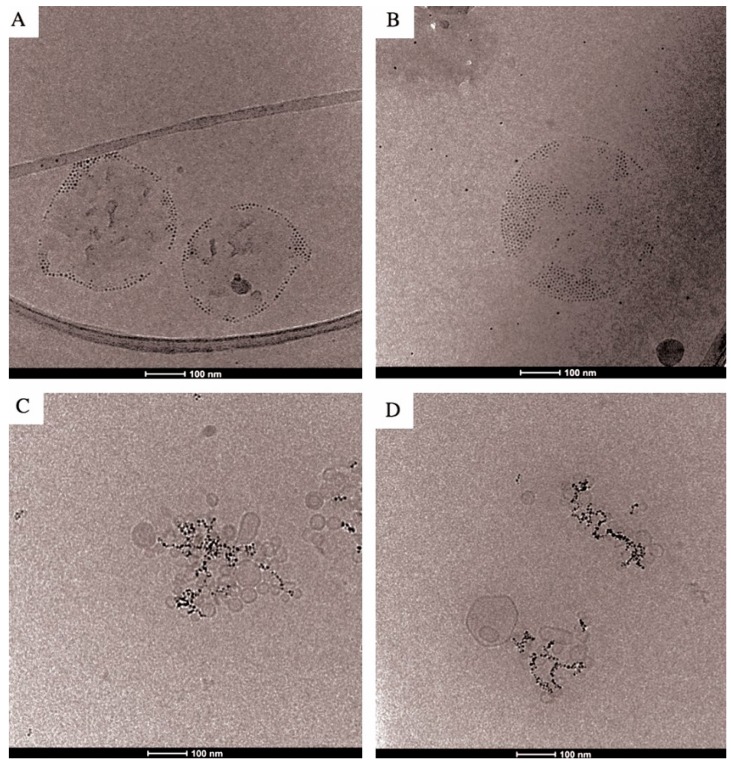
Cryo-TEM images of DMPC liposomes with hydrophobic (**A**,**B**) and hydrophilic (**C**,**D**) MNPs (scale bar: 100 nm). Liposomes are partially loaded with hydrophobic nanoparticles forming Janus-type nanoparticle-liposome hybrids (**A**,**B**). Hydrophilic particles are assembled forming branched (**C**) and lineal clusters (**D**).

Cryo-TEM was used to verify nanoparticle loading and its effect on liposomal structure (Figure 3). Figure 3A,B confirm that hydrophobic nanoparticles are incorporated into the lipid bilayer. The high contrast of magnetic nanoparticles made visualization of the bilayer extremely difficult. However, the disposition of the nanoparticles in circular structures not would be observed if nanoparticles were not embedded into the bilayer. It is known that nanoparticles can disrupt vesicle formation under certain conditions, for example, when the diameter of hydrophobic nanoparticles is well above the thickness of the bilayer. In such a case, the lipid of liposomes tends to adsorb around the nanoparticles resulting in micelle-like structures instead of vesicles [29,30]. Contrarily, if the nanoparticles were of smaller diameter, the adsorption of lipid around the particles would lead to an excessive curvature. To avoid this, nanoparticles embed into the bilayer. Other studies that have also incorporated iron oxide nanoparticles into the bilayers [31,32] shown this kind of interaction.

Theoretical studies have defined the mechanism of particle embedding as a result between the reduction of the energy of Gibbs obtained by moving a hydrophobic sphere from water into a hydrophobic milieu (Δ*G*_solv_) and the increase of energy of Gibbs produced in the deformation of the bilayer (Δ*G*_def_) [28]. In a system similar to the one used by us (nanoparticles of 5.5 nm of diameter and liposomes of dioleylphosphatidylcholine [29]) it was calculated that Δ*G*_solv_ is nearly an order of magnitude greater than Δ*G*_def_.

In our micrographs, the majority of nanoparticles are concentrated on one side of the vesicle giving some structures with appearance of Janus-type particles. This behavior has been also observed when hydrophobic gold nanoparticles were self-assembled with liposomes [33]. These authors indicated that the presence of nanoparticles in the bilayer results in a membrane deformation and separation, which establishes a void volume around the nanoparticle within the bilayer. Consequently, the nanoparticles cluster into the lipid bilayer in order to reduce the void space that surrounds them. This would explain the formation of the Janus-type nanoparticle vesicle-hybrid. Concerning the hydrophilic nanoparticles (Figure 3C,D), we observed that the size of liposomes encapsulating such nanoparticles is lesser than when liposomes were prepared with hydrophobic nanoparticles. The sonication step, although similar for both types of liposomes, produce vesicle populations of different sizes. According Michel et al. [34], in those systems formed by hydrophilic nanoparticles and fluid liposomes, where the interactions between membranes and nanoparticles are sufficiently attractive, when the radius of the particle is much smaller than the radius of the vesicle, the particle can either decorate the vesicle surface or be engulfed inside it. In the case of hydrophilic nanoparticles, their size is too small (below a critical radius that depends on the adhesion energy as well as on the value of the bilayer mean bending modulus) to be internalized inside the vesicles. We can observe that because the total adhesion energy does not overcome the energy needed for invagination, a large number of nanoparticles stay outside the liposome and form clusters. The obtained value of the hydrodynamic size for these liposomes corresponds indeed to the generated clusters—and not to the individual liposomes—as indicated by the obtained images.

### 2.2. Magnetic Resonance (MR) Contrast Properties

The MR contrast properties of the magnetoliposomes were evaluated in vitro using 0.5% agar phantoms (*T*_1_ ≈ 2630 ms and *T*_2_ ≈ 210 ms at 7 T) which simulate tumor tissues. The phantoms contained several types of hybrid nanoparticles (magnetoliposomes with different lipid composition and two different magnetic nanoparticles). The transversal (*T*_2_) relaxation times were measured with different amounts of magnetoliposomes. Although compounds bearing superparamagnetic nanoparticles are almost exclusively *T*_2_ contrast agents [35], for comparison, longitudinal (*T*_1_) relaxation times were also determined. Magnetic resonance relaxation behavior of water protons in the presence of contrast agent in phantoms was characterized by linear relationships between iron concentration and the inverse of proton relaxation times. The slopes of the straight lines indicated different longitudinal and transverse relaxivities. In this way, the relaxivities of non-liposomal magnetic nanoparticles were determined to be *r*_1_ = 0.96 mM^−1^·s^−1^ and *r*_2_ = 74.5 mM^−1^·s^−1^ (*r*_2_/*r*_1_ ≈ 78) for hydrophilic magnetic nanoparticles, and *r*_1_ = 0.80 mM^−1^·s^−1^ and *r*_2_ = 50.7 mM^−1^·s^−1^ (*r*_2_/*r*_1_ ≈ 63) for hydrophobic magnetic nanoparticles, the concentration being expressed in iron. It is well-known that the relaxivity ratio of *r*_2_/*r*_1_ is an important parameter to estimate the efficiency of *T*_2_ CA. The hydrophobicity/hydrophilicity of the coatings has an impact on the diffusion of water within the coating layer. Concerning the nanoparticles used, the hydrophilic ones afford relaxivities that are higher than those of hydrophobic ones. The presence of oleic acid excludes water molecules around the magnetic core, and, in consequence, extends the distance of water molecules from the core. As relaxivities are strongly affected by the distance between the aqueous medium and the magnetite core, the resulting relaxivities are lower.

It is difficult to compare these values with those reported for the commercial Resovist and Feridex, which are in the range of 7–14, since these values correspond to a magnetic field strength of 1.5 T [9]. Unlike the conventional paramagnetic contrast agents, i.e., gadolinium chelates, superparamagnetic nanoparticles have strong magnetic field strength dependency, and, for this reason, comparison of relaxivities must be always carried out at the same magnetic field strength. In this way, the values of relaxivities of the nanoparticles used in this study can be compared with those obtained at the same magnetic field strength with other superaparamagnetic nanoparticles employed by our group. In this way, magnetic particles of approximately 12 nm of diameter coated with polyethylenglycol of 4000 Da [36] gave the following values: *r*_1_ = 0.68 mM^−1^·s^−1^ and *r*_2_ = 311.1 mM^−1^·s^−1^; a commercial ferrofluid of similar size, stabilized with dextran (Micromod), gave *r*_1_ = 0.68 mM^−1^·s^−1^ and *r*_2_ = 337.4 mM^−1^·s^−1^. The highest *r*_2_ relaxivity values of these samples in comparison with the nanoparticles used in this study are due to the larger size of the magnetic core (12 nm vs. 5 nm), since the *T*_2_ contrast is enhanced not only by the magnetic field strength but also by the radius of iron oxide core [35]. In other words, the capability of MRI signal enhancement by nanoparticles correlates directly with the particle size [37].

Table 2 shows both types of relaxivities for the liposomal formulations used. As expected, *r*_1_ is poorly sensitive to concentration of iron oxide nanoparticles at 7 T due to the reduced susceptibility to dipolar contributions at high field, as well as the presence of bulky surface groups hindering the surface accessibility of water to the magnetic cores [38]. Contrarily, due to the superparamagnetic nature of magnetite cores, the transversal relaxivities are highly sensitive to the presence of any substances around the magnetic core.

**Figure 4:**
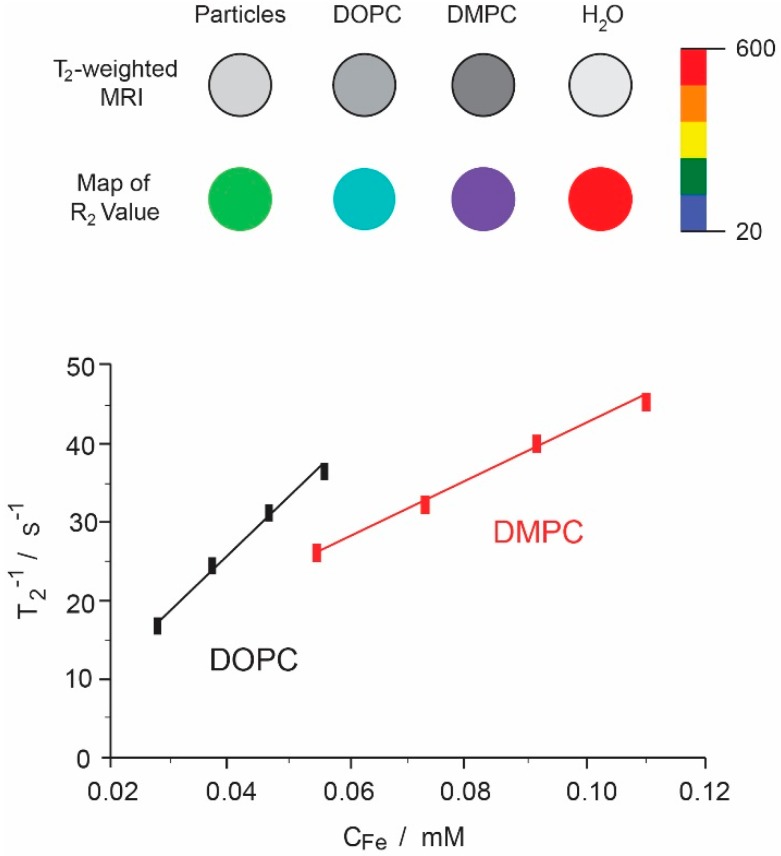
(**Upper**) *T*2 weighted contrast and color maps for iron oxide nanoparticles alone and in DOPC or DMPC liposomes; (**Lower**) Relaxation rates *T*_2_^−1^ as a function of iron concentration of magnetoliposomes formed by hydrophobic iron oxide nanoparticles and DOPC or DMPC.

Values of *r*_2_ are higher for all the as-synthesized formulations compared with magnetic nanoparticles alone. The chemical characteristic of any liposomal surface facilitates the adsorption of a water layer around the liposome and avoids the free diffusion of these molecules towards the magnetic core resulting in high relaxivity of liposomal formulations. The maximal value of *r*_2_ was obtained with liposomes of DMPC and hydrophilic nanoparticles. As we have observed in microscopic images, hydrophilic nanoparticles tend to cluster, reducing the access of water molecules to the nanoparticles’ surfaces and greatly increasing the microscale magnetic inhomogeneity of the sample. For this reason, the visualization of the effect of the different components will be limited to those formulations containing hydrophobic nanoparticles. First, we can observe that the lipid composition affects the MRI properties. Figure 4 shows the inverse of spin-spin proton relaxivities times for DMPC and DOPC liposomes containing the same hydrophobic nanoparticles. Relaxivity values of DOPC liposomes are almost 2-fold of those obtained with DMPC liposomes. In this case, the only difference between such formulations is the length of the lipid acyl chain and the degree of lipid saturation. In this way, the *r*_2_ relaxivity of liposomes with unsaturated phospholipids (DOPC) is higher compared to those with saturated lipids (DMPC). This fact can be due to the different accessibility of water to the magnetic core. The same tendency was observed when the formulation contained negative charge (PS) (Table 2). In general, the embedding of hydrophobic nanoparticles in liposomes leaded to an *r*_2_/*r*_1_ ratio higher than that obtained with non-embedded nanoparticles. As indicated previously, the nanoparticles become confined to a part of the bilayer (like a magnetic Janus-particle) so that each liposome acts as a highly magnetizable particle. After subjecting the particle to an external magnetic field, it is able to locally acquire a magnetic moment comparable to the sum of the magnetic moments of each individual encapsulated magnetic core. It is noteworthy to mention that magnetoliposomes with PS presented the highest values of *r*_2_/*r*_1_ ratio. We observed that the presence of PS in a liposomal formulation produced a reduction in size in comparison with the non-charged liposomes [39]. Assuming that this reduction is due to more compaction of the lipid chains, the movement of the water molecules will be also affected by the presence of PS. Finally, insertion of CHOL into the bilayer seems to reduce the relaxivity values. This tendency was also observed in a study using liposomes of soybean phosphatidylcholine and CHOL [40], and was also reported in magnetoliposomes of different lipid composition, but at lower magnetic field strengths [41]. Notwithstanding the above, considering the differences between the composition of the liposomes used in the above studies and the composition of those employed by us, it is probably not viable to make a direct comparison between these two kinds of formulations here and extract conclusions about the effect of CHOL.

### 2.3. Stability of Liposomal Samples

The size of the magnetoliposomes was determined after their incubation with isotonic saline or cell culture medium for 24 and 48 h. No significant differences were observed between the samples at room temperature and those kept at 4 °C. Magnetoliposomes incubated with culture medium aggregated. Their size after 24 h of incubation was almost double their value at time 0. The aggregation of pristine liposomes in the presence of serum components is a well-known common feature. It is widely proven that biomolecules, especially proteins, are attached to the surface of nanoparticles after their incubation with a biological milieu. These biomolecules form a dynamic shell known as protein corona [42]. However, for biomedical applications it is necessary that the surface coating of the nanoparticles presents anti-biofouling properties so that the nanoparticles are efficiently directed to the region of interest. The decoration of nanoparticles with polyethylene glycol (PEG) has been recognized as the strategy of choice, but recent studies have reported that PEGylation cannot entirely avoid the protein adsorption, although the degree of corona formation is undoubtedly diminished [43].

**Figure 5:**
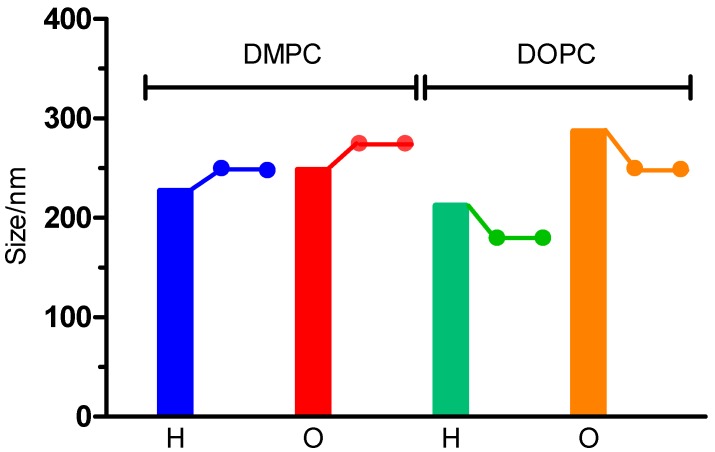
Change in the average diameter of four liposomal formulations after incubation with isotonic saline. The bar shows the size immediately after the mixing of the liposomes with saline. The value of the size after 24 and/or 48 h is marked with a dot. Magnetic hydrophilic (H) and hydrophobic (O) nanoparticles; DMPC: 1,2-Dimyristoyl-sn-glicerol-3 phosphatidylcholine; DOPC: 1,2-Dioleyl-sn-glicerol-3 phosphatidylcholine.

It is important to point out that magnetoliposomes with embedded hydrophobic nanoparticles present surface regions of a certain hydrophobicity (the hydrophobic part of any Janus-type nanoparticle), and such regions can attract very few plasma proteins. Hence, the liposome surface can be surrounded by a differential display of proteins depending on the presence or absence of hydrophobic nanoparticles in the bilayer. Magnetoliposomes incubated with isotonic saline did not show significant changes in their size (Figure 5). Average diameters remained unaltered after 24–48 h of incubation, which confirmed the good colloidal stability of the liposomes in the presence of sodium chloride.

## 3. Materials and Methods

### 3.1. Chemicals and Materials

All chemicals were reagent-grade and used without purification. SPIO magnetite nanoparticles (5 nm, 5 mg/mL) dispersed in toluene and SPIO magnetite nanoparticles (5 nm, 5 mg/mL) dispersed in water were purchased from Sigma-Aldrich (St. Louis, MO, USA). Based on the density of magnetite (5.1 g/cm^3^), 5 mg/mL is equivalent to 9.4 × 10^16^ particles/mL. CHOL, 1,2-Dimyristoyl-sn-glicerol-3 phosphatidylcholine (DMPC), and 1,2-Dioleyl-sn-glicerol-3 phosphatidylcholine (DOPC) were purchased from Sigma-Aldrich (St. Louis, MO, USA). Phosphatidylserine (PS) was purchased from Lipid Products (Redhill, Surrey, UK). Ultrapure water at 18.2 mΩ was obtained from a Millipore purification system (Millipore, Bedford, MA, USA) and used in all experiments.

### 3.2. Preparation of Magnetoliposomes

Magnetoliposomes (MLs) were prepared according Chen et al. [44]. The lipid compositions were DMPC, DMPC:CHOL (2:1, molar ratio), DMPC:PS (9:1, molar ratio), DOPC, DOPC:CHOL (2:1, molar ratio), and DOPC:PS (9:1, molar ratio), at 10 mM lipid concentration. The lipid molecule to nanoparticle (L/N) ratio was approximately of 10,000:1. For obtaining hydrophobic magnetoliposomes (O-MLs), lipid (367 μL) and hydrophobic magnetic nanoparticles (402 μL) were mixed and placed in a round-bottom flask. Organic solvents were removed by rotary evaporation at 37 °C and reduced pressure for 45 min. Once the solvent was removed, the resulting lipid/nanoparticles film was hydrated with 1 mL of water. For hydrophilic magnetoliposomes (H-MLs), lipid (367 μL) was previously evaporated, and afterwards, the lipid was hydrated with 1 mL of an aqueous suspension of hydrophilic suspension of magnetic nanoparticles (2.01 mg/mL). After complete hydration, both suspensions (O-MLs and H-MLs) were sonicated in two steps. First, a gentle sonication in a bath sonifier (Transonic Digitals, Elma, Germany) for 30 min, and, then, five sonication steps of 10 s carried out in an UP200St ultrasonic processor (Hielscher, Teltow, Germany). The separation of liposome-encapsulated and free SPIONs was accomplished by gel exclusion chromatography (GEC). To this aim, 250 μL of MLs were applied to a 2.5-mL syringe filled with Sepharose 4B CL. After elution with water, MLs were separated from non-encapsulated ferrofluid. To exclude the possibility that hydrophilic SPIONs aggregate and co-elute with liposomes during GEC, free SPION dispersions were treated by the identical procedure used for ML formation (without addition of lipids) and assessed by GEC.

### 3.3. Characterization

The hydrodynamic diameter and the corresponding polydispersity index (PI) of MLs were determined by dynamic light scattering at a fixed scattering angle of 90° with a Zetasizer Nano (Malvern, UK) at 25 °C. MLs from the stock solution were dispersed in water to obtain approximately 0.1 g·L^−1^ solid content. Geometry of MLs was observed by transmission electron microscopy (TEM) and cryo-TEM. For TEM observations, a Jeol 1010 microscope (Jeol, Tokyo, Japan) operating at 80,000 kV was used. Samples were prepared by placing a drop of MLs onto a 400-mesh copper grid coated with carbon, and after staining with uranyl acetate they were allowed to dry in the air before placing into the microscope. Images were recorded with a Megaview camera. Acquisition was accomplished with the Soft-Imaging software (SIS, Münster, Germany). For cryo-TEM observations, grids were transferred to a Tecnai F20 (FEI, Eindhoven, The Netherlands) using a cryoholder (Gatan, Warrendale, PA, USA). Images were taken at 200 kV, at a temperature ranging from −175 to −170 °C and using low-dose imaging conditions with a 4096 × 4096 pixel CCD Eagle camera (FEI, Eindhoven, The Netherlands).

### 3.4. Quantification of Iron and Lipid Content

The iron content of MLs was determined by visible spectrophotometry on the basis of the ferrous ion using *o*-phenanthroline [45]. The calibration curve was performed with a solution of Fe_3_O_4_ (Aldrich, Milwaukee, WI, USA) in 12 mmol·L^−1^ HCl. The phospholipid content was determined by the Steward-Marshall method [46]. The calibration curve was performed with the same different lipid mixtures in chloroform that the used in the study. Absorbance was measured in a Shimadzu UV-2401 PC UV-vis spectrophotometer (Shimadzu, Kyoto, Japan).

### 3.5. Determination of the Relaxivity

MRI experiments were conducted on a 7.0 T BioSpec 70/30 horizontal animal scanner (Bruker BioSpin, Ettlingen, Germany). *T*_1_ relaxometry maps were acquired by using RARE (rapid acquisition with rapid enhancement) sequence applying nine repetition times. For the *T*_2_ relaxometry maps, MSME (multi-slice multi-echo) sequence was used with a repetition time = 4764.346 ms with 16 echo times. The relaxation rates, *R*_1_ = 1/*T*_1_ and *R*_2_ = 1/*T*_2_ for each sample were processed using the Paravision 5.1 software (Bruker, BioSpin, Ettlingen, Germany). The relaxivity for a MRI CA is defined as the increase of the relaxation rate of the solvent (water) induced by 1 mmol·L^−1^ of the active ion and it is calculated according to Equation (4):

(4) $$r_{i\left(obs\right)}=\left[\frac{1}{T_{i\left(obs\right)}}-\frac{1}{T_{i\left(water\right)}}\right]/c_{Fe}$$

Herein, *i* = 1 or 2, and *c*_Fe_ is the analytical iron concentration as determined by the *o*-phenanthroline reaction. The relaxivities were computed using linear regression analysis to fit relaxation rates and molar iron concentrations.

To avoid particle aggregation during the period in which the magnetic field is applied, MLs and SPIONs were prepared by diluting them in air bubble-free agar phantoms. Ultra-pure agar solution was made at 75 °C. To remove air bubbles, nitrogen gas was flushed through the agar, and vacuum suction was applied. Then, the suspension was allowed to cool slowly to approximately 37 °C, and agar was mixed with MLVs or ferrofluid, and the mixing was placed in a 96-wells culture plate. The iron concentrations ranged from 0.17 to 0.49 mM.

### 3.6. Stability of Liposomal Samples

The stability of several liposomal samples over time was evaluated by determining the change in their hydrodynamic diameter for a period of 24–48 h. Aliquots (50 μL) of purified DOPC- and DMPC-liposomes containing either hydrophobic or hydrophilic nanoparticles were diluted with 3 mL of isotonic saline or cell culture medium (Dulbecco’s modified Eagle Medium (DMEM) with 10% fetal calf serum) and kept at room temperature and in a freezer in quiescent conditions. 

## 4. Conclusions

We have characterized superparamagnetic liposomes with saturated DMPC and unsaturated DOPC phospholipids, and with or without CHOL or PS. The incorporation of hydrophilic or hydrophobic magnetic particles in the DMPC- or DOPC-based liposomes resulted in hybrid structures with higher transverse relaxation rates (*r*_2_) compared with naked magnetic nanoparticles. The main reason for this was that the diffusion coefficient of water near the nanoparticles was reduced. The liposomal coating thus ensured a longer interaction between the water protons and the magnetic field at the surface of the magnetic core than in absence of coating. Cryogenic electron transmission microscopy revealed effective embedding of hydrophobic magnetic particles into the bilayer, producing asymmetric structures similar to Janus-type nanoparticles, since nanoparticles were observed to cluster in a part of the bilayer. This finding renders cluster-liposome hybrids particularly promising candidates for MRI applications. In regards to the hydrophobic nanoparticles, the relaxivity of liposomes with unsaturated phospholipids was higher than that of those with saturated lipids. CHOL led to a smaller reduction in relaxivity. The highest relaxivity was obtained for magnetoliposomes containing PS.

In general, it is feasible to use magnetoliposomes as negative contrast agents for MRI, making these nanosystems good candidates to be taken into consideration for molecular imaging. The contrast is particularly enhanced when hydrophobic nanoparticles are used, since, in this case, due to the uniform size and high crystallinity of the nanoparticles, there was an additional clustering effect into the bilayer. Because of these facts, high values of *r*_2_ are obtained. Furthermore, the diffusivity of the water in the coating can be modulated by changing the liposome composition (charge, degree of saturation, length of the hydrocarbon chain, and presence or absence of CHOL). These hybrid structures can be administered intravenously and due to the absence of steric stabilizers, e.g., PEG, they are metabolized by cell of the mononuclear phagocyte system (MPS), accumulating in liver, spleen, and bone marrow. In this way, these magnetoliposomes can be used for tumor detection, especially in liver diseases.

Magnetic nanoparticles decrease the magnetic resonance signal intensity due to the uptake by kupffer cells. Therefore, the image of the tissue appears dark. However, tumor lesions reduce the uptake and the image of the tissue appears bright relative to the surrounding tissue. Thus, magnetic particles produce a strong contrast between normal and abnormal liver, thereby enabling clear detection of the abnormal tissue.

## Figures and Tables

**Table 1:** Encapsulation efficiency, average of cross-sectional area, and number (*N*) of magnetic hydrophilic (H) and hydrophobic (O) nanoparticles encapsulated into liposomes of different lipid composition. MLs: Magnetoliposomes; DMPC: 1,2-Dimyristoyl-sn-glicerol-3 phosphatidylcholine; DOPC: 1,2-Dioleyl-sn-glicerol-3 phosphatidylcholine; CHOL: Cholesterol and PS: Phosphatidylserine.

MLs	Encapsulation Efficiency/μmol Magnetite	Average Cross-Sectional Area/nm^2^	*N*
H-DMPC	6.9	0.59	3
O-DMPC	43.6	0.59	16
O-DMPC-CHOL	22.8	0.51	11
O-DMPC-PS	20.4	0.63	10
H-DOPC	7.9	0.72	2
O-DOPC	274.0	0.72	17
O-DOPC:CHOL	49.0	0.60	15
O-DOPC-PS	17.7	0.71	9

**Table 2:** *r_1_*, *r*_2_ and *r_2_*/*r_1_* *ratio* of magnetic hydrophilic (H) and hydrophobic (O) nanoparticles encapsulated into liposomes of different lipid composition. MLs: Magnetoliposomes.

MLs	*r_1_/*mM^−1^·s^−1^	*r*_2_/mM^−1^·s^−1^	*r*_2_/*r*_1_
H-DMPC	9.1	1282	140
O-DMPC	0.9	340	378
O-DMPC-CHOL	0.8	230	288
O-DMPC-PS	0.8	798	~1000
H-DOPC	3.4	678	199
O-DOPC	0.9	630	700
O-DOPC:CHOL	0.9	281	312
O-DOPC-PS	0.9	995	~1000
